# Guidelines for the Diagnosis, Treatment and Prevention of Postoperative Infections

**DOI:** 10.1155/S1064744903000097

**Published:** 2003

**Authors:** John W. Larsen, W. David Hager, Charles H. Livengood, Udo Hoyme

**Affiliations:** 1 Department of Obstetrics and Gynecology The George Washington University 2150 Pennsylvania Avenue Washington DC NW 20037 USA; 2 Department of Obstetrics and Gynecology University of Kentucky School of Medicine Lexington KY USA; 3 Department of Obstetrics and Gynecology Duke University Medical Center Durham NC USA; 4 Frauenklinik Erfurt Erfurt Germany

## Abstract

Bacterial contamination of the operative site is a common occurrence in obstetrics and gynecology. The widespread use of antibiotic prophylaxis has reduced but not eliminated serious postoperative infections. For most operations, a single dose of a limited-spectrum drug has been as effective as a multidose regimen. In the differential diagnosis it is important to consider cellulitis, abscess, necrotizing fasciitis and septic pelvic thrombophlebitis. Abscess and necrotizing fasciitis are expected to require invasive therapy in addition to antibiotics, while cellulitis and septic pelvic thrombophlebitis should respond to medical management alone. Although a postoperative fever is a warning sign of possible infection, it may also be caused by the antibiotics that are given for treatment. The use of prolonged courses of antibiotics once the patient is clinically well is discouraged. While clinical guidelines are provided for use in the diagnosis and management of postoperative infections, these recommendations are intended for general direction and not as an exclusive management plan.


*Infect Dis Obstet Gynecol 2003;11:65–70*

### Guidelines for the diagnosis, treatment and prevention of postoperative infections

**John W. Larsen[1], W. David Hager[2], Charles H. Livengood[3] and Udo Hoyme[4]**

[1]*Department of Obstetrics and Gynecology, The George Washington University, Washington, DC*
[2]*Department of Obstetrics and Gynecology, University of Kentucky School of Medicine, Lexington, KY*
[3]*Department of Obstetrics and Gynecology, Duke University Medical Center, Durham, NC*
[4]*Frauenklinik Erfurt, Erfurt, Germany*

Bacterial contamination of the operative site is a common occurrence in obstetrics and gynecology. The widespread use of antibiotic prophylaxis has reduced but not eliminated serious postoperative infections. For most operations, a single dose of a limited-spectrum drug has been as effective as a multidose regimen. In the differential diagnosis it is important to consider cellulitis, abscess, necrotizing fasciitis and septic pelvic thrombophlebitis. Abscess and necrotizing fasciitis are expected to require invasive therapy in addition to antibiotics, while cellulitis and septic pelvic thrombophlebitis should respond to medical management alone. Although a postoperative fever is a warning sign of possible infection, it may also be caused by the antibiotics that are given for treatment. The use of prolonged courses of antibiotics once the patient is clinically well is discouraged.

While clinical guidelines are provided for use in the diagnosis and management of postoperative infections, these recommendations are intended for general direction and not as an exclusive management plan.

Key words: ABSCESS; ANTIBIOTICS; CELLULITIS; FASCIITIS; FEVER; THROMBOPHLEBITIS

### BACKGROUND

Most operations in obstetrics and gynecology involve some degree of bacterial contamination, and are classified as 'clean-contaminated' cases, even when the patient has no preoperative symptoms of active infection[1]. Prior to the current practice of routine prophylactic antibiotics, postoperative infections were common (range 5–10%, depending on the type of surgery and clinical risk factors). The widespread use of antibiotic prophylaxis has reduced but not eliminated serious postoperative infections, the average expected wound infection rate being 4% in gynecology and 6% after Cesarean sections. These rates are increased in the presence of other risk factors, such as gross contamination of the operative site, massive obesity or prolonged operative time.

This article will offer recommendations based on existing scientific data, theoretical rationale, and the clinical experience of the authors and other members of the Infectious Disease Society for Obstetrics and Gynecology. Our recommendations are scored according to a modified Centers for Disease Control (CDC) system for categorizing recommendations[1].

(1) Category IA. Strongly recommended for implementation and supported by well

Correspondence to: John W. Larsen, MD, The George Washington University, 2150 Pennsylvania Avenue, NW, Washington, DC 20037, USA. E-mail: jlarsen@mfa.gwu.edu

© 2003 The Parthenon Publishing Group designed experimental, clinical or epidemiological studies.

(2) Category IB. Strongly recommended for implementation and supported by suggestive clinical or epidemiological studies or theoretical rationale.

(3) Category II. Suggested for implementation and supported by suggestive clinical or epidemiological studies or theoretical rationale.

(4) Category III. Opinions based on clinical experience, descriptive studies or expert committees.

The category scoring system will be shown in parentheses throughout the text.

### PROPHYLAXIS

Any antibiotic that has a spectrum of effectiveness against the bacteria that most often contaminate surgical sites is likely to reduce the incidence of postoperative infection. However, it is not necessary for the antimicrobial agent used for prophylaxis to cover each and every possible pathogen. For most gynecological operations and Cesarean sections, a single prophylactic dose of a limited-spectrum drug, such as cefazolin, has been shown to be as effective as a multidose regimen or one that uses more costly, wider-spectrum agents[2,3]. For gynecological procedures, the dosing of antimicrobial prophylaxis should occur no more than 30 minutes before the surgery[1]. For Cesarean sections, the dosing may begin at cord clamping, unless there is earlier timing as part of prophylaxis for Group B *Streptococcus*[1,3,4] (category IA).

### ABDOMINAL WOUND INFECTIONS

The least severe postoperative infections of the abdominal wall are localized to the skin and fatty tissue above the fascia. If there is cellulitis alone, the only manifestations may be redness, warmth and swelling with some degree of localized pain and tenderness. If there is no fluid drainage from the wound, no hematoma and the edges are intact, antimicrobial treatment of the cellulitis will often be sufficient. A very small amount of serous drainage from the wound may be managed without opening the wound, provided that there is no other evidence of serious infection or wound disruption. If there is purulent drainage from the wound, it should be opened widely to allow drainage of pus and removal of necrotic tissue. The wound should be probed gently to check for fascial integrity. If the fascia is intact, the healing process is hastened by mechanical debridement followed by loose packing of the wound with gauze moistened by saline. The wet gauze dressings should be changed two to three times daily. Dilute hydrogen peroxide (a 1:1 mixture of hydrogen peroxide and saline) may be used at the time of dressing changes to facilitate gentle removal of exudate. Some experts prefer Dakin's solution, particularly if the necrotic tissue is relatively abundant and/or persistent. Irrigation with povidone–iodine solution is not advised[5-7] (category III).

A deep wound infection will involve the fascia and muscle. The fascia may split open, leading to evisceration or ventral hernia. The most severe form of deep wound infection is necrotizing fasciitis (NF), in which infection by synergistic aerobic and anaerobic bacteria leads to necrosis of all layers. NF is likely to be fatal unless prompt wide surgical debridement is performed. Early NF is indistinguishable from cellulitis on examination. Later NF is manifested by severe pain, and clinical signs and symptoms of sepsis, as well as a wound drainage that is thin and may appear like dirty dishwater rather than viscid pus. Manifestations may be particularly fulminant in diabetics and immunocompromised patients. The skin may show bullae, and crepitus may be present to palpation. The diagnosis of necrotizing fasciitis is confirmed by surgical exploration of the wound, which reveals necrotic (non-bleeding, discolored and decomposing) subcutaneous tissue and fasciae. When managing necrotizing fasciitis, all necrotic tissue must be removed while maintaining broad-spectrum antibiotic coverage (Table 1). The resection should be extensive at the first attempt, rather than relying on a gradual, bit-by-bit approach. Nonetheless, if the infection-related necrosis continues to spread, the surgical debridement must be repeated[5–7] (category IB).

Systemic manifestations due to wound infections may be relatively minimal in truly localized

**Table 1** Intravenous antibiotic regimens for treating postoperative infections

| *Category* | *Agent* |
|---|---|
| Localized infection with minimal systemic findings | Cefotaxime, 1.0 g every 8 hours<br>Cefotetan, 2.0 g every 12 hours<br>Cefoxitin, 2.0 g every 6 hours<br>Ceftriaxone, 2.0 g followed by 1.0 g every 24 hours<br>Piperacillin, 4.0 g every 6 hours<br>Ampicillin/sulbactam, 3.0 g every 6 hours<br>Mezlocillin, 4.0 g every 6 hours<br>Ticarcillin/clavulanic acid, 3.1 g every 4–6 hours |
| Extensive infection with moderate to severe systemic findings | Clindamycin, 900 mg IV every 8 hours<br>*plus*<br>Gentamicin, 2.0 mg/kg IV followed by 1.5 mg/kg IV every 8 hours<br>Alternatively, gentamicin may be given as a single daily dose of 5 mg/kg<br>Ampicillin, penicillin or vancomycin may be added to cover enterococci.<br>Vancomycin-resistant enterococci are being reported. If vancomycin-resistant enterococci are proved to be the cause of a serious infection, treatment with linezolid (Zyvox) or quinupristin/dalfopristin (Synercid) may be necessary<br>*or*<br>Ampicillin, 2.0 g IV followed by 1 g IV every 4 hours<br>*plus*<br>Gentamicin, 2.0 mg/kg IV followed by 1.5 mg/kg IV every 8 hours<br>*plus*<br>Metronidazole, 500 mg IV every 8 hours<br>*or*<br>Imipenem/cilastatin, 500 mg to 1000 mg IV every 6 hours<br>*or*<br>Levofloxacin 500 mg IV every 24 hours<br>*plus*<br>Metronidazole 500 mg IV every 8 hours |

infections, progressing to fever and then to septic shock as the infection becomes more extensive. Cellulitis is usually treated with a single agent that is effective against streptococci, staphylococci and most Gram-negative aerobes (Table 1). If pus has accumulated or there is a deep infection extending into the pelvis, anaerobic bacteria should be covered from the outset of antimicrobial therapy. Antimicrobial therapy should continue until the patient has been afebrile for 24–48 hours. If septic shock is present, debridement and antibiotic therapy must be accompanied by vigorous medical support of other damaged organ systems[7,8] (category IB).

### PELVIC CELLULITIS AND ABSCESS

Although inflammation is normal at the operative site as wound healing proceeds, the clinician must remain alert to diagnose and treat if pelvic cellulitis or abscess develops. Pelvic cellulitis often presents with an increase in low abdominal pain, increased vaginal discharge exuding from the vaginal cuff and a low-grade fever. Pelvic examination and possible ultrasound will show no mass. Treatment should be with a single agent as shown in Table 1. Oral antibiotics may follow initial parenteral treatment until the patient has been afebrile for 24–48 hours (category IA).

If pelvic examination and ultrasound or computed tomography scan reveal a fluid collection at the operative site in a symptomatic patient, this is usually an infected hematoma or cuff abscess. Mechanical drainage should be established in addition to using antibiotic therapy. Under conscious sedation or light general anesthesia, drainage is usually established by bluntly probing the vaginal cuff area to break up adhesions, thus permitting egress of pus and old hematoma contents. Drainage via a needle or catheter guided by ultrasound or CT scan is also an option[9] (category II).

### PUERPERAL ENDOMETRITIS

Puerperal endometritis is a relatively common complication of Cesarean section, particularly if there was an amniotic fluid infection prior to delivery. It is manifested as fever (temperature ≥ 38°C on two or more occasions after the first 24 hours postpartum or > 38.5°C at any time) and uterine tenderness, progressing to clinical sepsis in the more severe cases. The clinician should be alert for development of abscess formation if there is an infected hematoma in the bladder flap or broad ligament(s). Antimicrobial treatment should include coverage of anaerobes from the outset, and should continue until the patient has been afebrile and asymptomatic for 24–48 hours[8,9] (category IA).

### SEPTIC PELVIC THROMBOPHLEBITIS

Septic pelvic thrombophlebitis should be considered in the postpartum or postoperative female patient in whom fever continues after appropriate parenteral antibiotic therapy has been administered for at least 72 hours. In addition to the classic signs of infection, the patient may present with upper thigh pain, and with edema and tenderness in the lower extremity if iliofemoral venous thrombosis is present. If there is thrombosis of the ovarian vein, a tender cord may be palpable on abdominal examination. In the past, the diagnosis of septic pelvic thrombophlebitis was implied by the absence of localizing signs of infection and a successful response to heparin therapy. Currently, the diagnosis is often confirmed by computed tomography scan or magnetic resonance imaging[10,11] (category II).

The treatment of septic pelvic thrombophlebitis is medical, not surgical. In many cases the patient will continue to improve if antibiotic therapy is continued (perhaps with a change in agents used) without anticoagulant therapy[11]. The other option is to continue full antibiotic coverage of aerobic and anaerobic bacteria (Table 1) while adding intravenous heparin until the patient has been afebrile and clinically well for 24–48 hours. Lysis of fever may occur within 24–48 hours after starting heparin, while other cases may require more prolonged therapy for full resolution. Although it has been traditional to continue heparin for 5–7 days after defervescence of the fever, the heparin should only be continued until the patient is afebrile and clinically well. Conversion to oral anticoagulants is not necessary[11] (category II).

### GENERAL DISCUSSION

The diagnosis of postoperative infection is generally made when there is:

(1) pain and tenderness in the area contiguous with the infection;

(2) an oral temperature of ≥ 38°C on two separate occasions at least 6 hours apart, or of > 38.5°C at any time.

In cases of surgical wound infection, skin erythema and/or subcutaneous induration and/or incisional drainage are usually present. In cases of pelvic cellulitis or abscess, extensive induration or a mass, respectively, are usually found on rectovaginal examination. The diagnosis may be supported by leukocytosis of > 13 000/mm$^3$ with > 90% bands plus polymorphonuclear leukocytes. Delayed signs include a positive blood culture or positive culture from the wound, operation site or abscess cavity.

We urge that, whenever possible, efforts be made in each case to distinguish infection from inflammation. It is particularly important when undertaking infection research to use clear criteria for the inclusion and exclusion of infection, thereby adding to the value of any study. Nonetheless, in clinical practice it is recognized that in many cases it may be necessary to treat empirically when signs and symptoms of infection are mild, in order to protect patient well-being. With localized wound infections, drainage alone may be sufficient for cure (category II). If an abscess or fluid collection is detected, it is usually necessary to establish drainage in order to achieve resolution. Antibiotic treatment is usually given concomitantly. Alternatively, antibiotic treatment may be withheld temporarily until the pus has been collected for culture (category III).

The choice of which antibiotic(s) to use continues to evolve. The scope of treatment is usually directed to cover endogenous bacteria. In most cases, multiple bacterial species are involved, including both aerobic and anaerobic organisms. In many cases, clinical health will be restored even if the antibiotic treatment covered some but not all of the bacteria present in the infected area. Since it is extremely costly and time-consuming to culture all anaerobic bacteria, these studies are generally not undertaken, and therapy must be selected before culture results are available in the great majority of cases. The agent used for prophylaxis generally should not be used for therapy. However, it may be acceptable as part of a combination of drugs in a treatment regimen, particularly if the prophylaxis was given as a single dose before the emergence of symptoms (category IB).

If treatment is effective, defervescence of pyrexia usually occurs within 48–72 hours. If defervescence has not occurred, then a review of cultures and sensitivities and a switch of antibiotics are usually recommended. An exhaustive search for abscess, remote infection, unrecognized visceral injury and non-infectious causes of fever should be undertaken. 'Drug fever' should be considered probable in a patient who has been treated with parenteral antibiotics yet remains febrile, has no localizing signs of infection, and has eosinophilia on her differential white blood cell count. In this situation, antibiotics should be discontinued while the patient is observed for defervesence of the fever. If pyrexia continues, heparin should be considered for empirical treatment of possible septic thrombophlebitis (category II).

The standard duration of antibiotic treatment is until the patient has been afebrile and asymptomatic for 24–48 hours. Current guidelines for acute-care hospitalization are evolving towards shorter hospital stays with flexible treatment guidelines for the conclusion of therapy in patients who are generally healthy and who are likely to be compliant with outpatient treatment. Either intravenous or oral antibiotics may be used to complete therapy at home if the patient is considered to be nearly cured as demonstrated by decreasing fever to nearly normal and no evidence of an unresolved abscess. On the other hand, oral antibiotics are not needed after the patient has responded to parenteral antibiotic therapy, and is afebrile and clinically well[12]. Prolonged courses of antibiotics for patients who are afebrile and asymptomatic are discouraged in order to minimize adverse reactions and the development of resistant bacteria (category IA).

### ACKNOWLEDGEMENTS

This paper was reviewed by members of the Infectious Disease Society for Obstetrics and Gynecology.

### REFERENCES

1. Mangram AJ, Horan TC, Pearson ML, *et al.* Guideline for prevention of surgical site infection, 1999. *Infect Control Hosp Epidemiol* 1999;20: 247–80
2. Mittendorf R, Aronson MP, Berry RE, *et al.* Avoiding serious infections associated with abdominal hysterectomy: a meta-analysis of antibiotic prophylaxis. *Am J Obstet Gynecol* 1993; 169:1119–24
3. Carlson C, Duff P. Antibiotic prophylaxis for cesarean delivery: is an extended-spectrum agent necessary? *Obstet Gynecol* 1990;76:343–6
4. Hager WD, Schuchat A, Gibbs R, *et al.* Prevention of perinatal group B streptococcal infection: current controversies. *Obstet Gynecol* 2000;96: 141–5
5. Stone H, Martin JD. Synergistic necrotizing cellulitis. *Ann Surg* 1972;175:702–11

6. Stephenson H, Dotters DJ, Katz V, *et al*. Necrotizing fasciitis of the vulva. *Am J Obstet Gynecol* 1992;166:1324–7
7. Sudarsky LA, Laschinger JC, Coppa GF, *et al*. Improved results from a standardized approach in treating patients with necrotizing fasciitis. *Ann Surg* 1987;206:661–5
8. diZerega G, Yonekura L, Roy S, *et al*. A comparison of clindamycin–gentamicin and penicillin–gentamicin in the treatment of post-cesarean section endometritis. *Am J Obstet Gynecol* 1979; 134:238–42
9. Brumfield CG, Hauth JC, Andrews WW. Puerperal infection after cesarean delivery: evaluation of a standardized protocol. *Am J Obstet Gynecol* 2000;182:1147–51
10. Brown CEL, Lowe TW, Cunningham FG, *et al*. Puerperal pelvic thrombophlebitis: impact on diagnosis and treatment using X-ray computed tomography and magnetic resonance imaging. *Obstet Gynecol* 1986;68:789–94
11. Brown CE, Stettler RW, Twickler D, *et al*. Puerperal septic pelvic thrombophlebitis: incidence and response to heparin therapy. *Am J Obstet Gynecol* 1999;181:143–8
12. Hager WD, Pascuzzi M, Vernon M. Efficacy of oral antibiotics following parenteral antibiotics for serious infections in obstetrics and gynecology. *Obstet Gynecol* 1989;73:326–9

RECEIVED 07/09/02; ACCEPTED 09/24/02
